# Surface Plasmon Resonance (SPR)- and Localized SPR (LSPR)-Based Virus Sensing Systems: Optical Vibration of Nano- and Micro-Metallic Materials for the Development of Next-Generation Virus Detection Technology

**DOI:** 10.3390/bios11080250

**Published:** 2021-07-26

**Authors:** Kenshin Takemura

**Affiliations:** Sensing System Research Center, The National Institute of Advanced Industrial Science and Technology, 07-1 Shuku-Machi, Tosu 841-0052, Japan; takemura.kenshin@aist.go.jp; Tel.: +81-942-81-3619

**Keywords:** surface plasmon resonance, localized SPR, biosensing, virus sensing, micro-scale, nanoscale

## Abstract

The global damage that a widespread viral infection can cause is evident from the ongoing COVID-19 pandemic. The importance of virus detection to prevent the spread of viruses has been reaffirmed by the pandemic and the associated social and economic damage. Surface plasmon resonance (SPR) in microscale and localized SPR (LSPR) in nanoscale virus sensing systems are thought to be useful as next-generation detection methods. Many studies have been conducted on ultra-sensitive technologies, especially those based on signal amplification. In some cases, it has been reported that even a low viral load can be measured, indicating that the virus can be detected in patients even in the early stages of the viral infection. These findings corroborate that SPR and LSPR are effective in minimizing false-positives and false-negatives that are prevalent in the existing virus detection techniques. In this review, the methods and signal responses of SPR and LSPR-based virus detection technologies are summarized. Furthermore, this review surveys some of the recent developments reported and discusses the limitations of SPR and LSPR-based virus detection as the next-generation detection technologies.

## 1. Introduction

The current COVID-19 pandemic caused by SARS-CoV-2 has revealed how highly infectious emerging viruses spread in modern society and the gravity of social consequences such infections can cause [1,2,3]. Viral infections can spread rapidly, especially via droplet transmission, as in the case of SARS-CoV-2 and similar viruses [4]. Nowadays, where people travel around the world with much ease, viruses capable of spreading via droplet transmission are one of the most dangerous causes of infectious diseases. Outbreaks of emerging viruses have been attributed mainly to adaptive outbreaks [5,6]. Genetic mutations that allow infection of previously uninfected hosts can lead to explosive pandemics [7]. These facts imply that emerging viral outbreaks and pandemics will continue to occur with high transmissibility. One way to prevent or control such pandemics is to develop a virus detection technology that enables the prevention of the global spread of viruses.

Polymerase chain reaction (PCR)-based detection methods form the gold standard methods for virus detection [8]. However, the cost of equipment, the need for skilled technicians, and the time required for detection are their limitations [9]. For the SARS-CoV-2 pandemic, the problem was almost solved by expanding the number of sites with PCR facilities and active research and development worldwide [10,11,12]. In recent years, the shortage of skilled personnel, which is the most important limitation of PCR, is also being solved by automating the PCR system to detect the extracted viral RNA [13,14]. Therefore, next-generation technologies should have a reliable single-step detection of the target virus and require minimum time for detection.

On the surface of a solid material such as a metal with a free charge, the surface charge (mostly electrons) oscillates collectively due to light irradiation. This phenomenon is called surface plasmon resonance (SPR). [15]. SPR-induced surface charge oscillations are coupled with electromagnetic waves under certain conditions. The quantum of these oscillations is described as the surface plasmon polariton (SPP), and the excitation of SPP is an essential step in SPR biosensors [16,17,18]. SPR significantly increases the surface sensitivity of the substrates to react with the target material in certain spectroscopic measurement techniques, as characterized by the conditions of resonance between the irradiated light and the substrate [19,20]. The increased sensitivity of SPR has also been applied in virus detection techniques [21,22,23,24,25]. Compared to SPR on an individual substrate such as a thin metal film, the plasmon phenomenon similarly produced by irradiating light on metal nanoparticles is described as localized SPR (LSPR) [26]. The plasmon phenomenon generated on the nanoparticle surface generates a strong electric field in the vicinity of the nanoparticles [27]. Within this plasmon region, the interaction of light with molecules and other types of fluorescent nanomaterials is enhanced [28]. Furthermore, when nanoparticles that form an electric field are in close proximity to each other or bind together, a significantly enhanced electric field is formed between the particles, and in this electric field, Raman scattering of certain chemical species is electromagnetically enhanced [29]. Surface-enhanced infrared absorption (SEIRA) has also been reported as an LSPR-based application [30,31]. The potential for application in biosensing is high, because infrared absorption can be induced in the infrared region by adjusting the shape and size of the nanoparticles. [32]. The plasmon resonance effect can be applied to various biosensing fields because it produces a strong optical response and signal enhancement at the micro/nanoscale.

**Figure 1:**
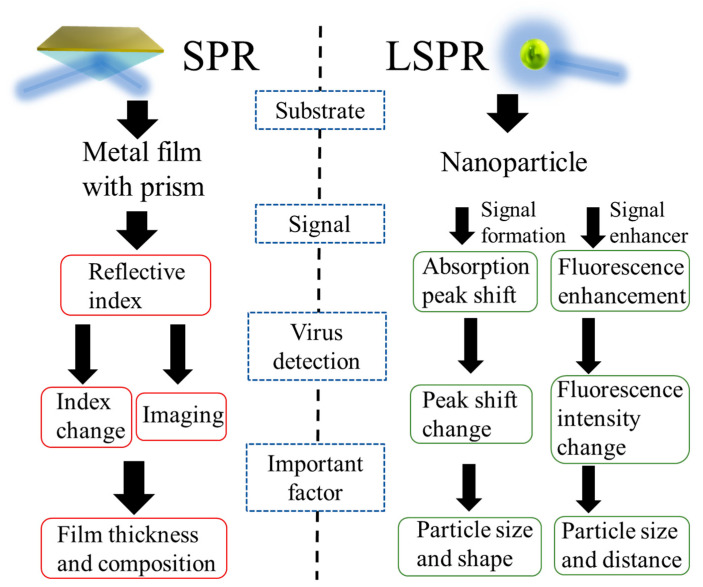
Diagram summarizing the foundation, signals to be used, signal measurement methods, and critical factors as building elements for a virus detection system using SPR and LSPR.

In this review, virus detection techniques and their performances based on signal amplification characteristics assessed by SPR and LSPR on a micro/nanoscale basis are explained. SPR uses as a signal the optical changes that occur when a virus is coupled to a sensor that has a prism bonded to a metal film to couple the light. For LSPR, the strong plasmons generated locally on the nanoparticles are used as a signal or as an enhancer for intensity of fluorescence material (Figure 1). In SPR-based detection systems, the properties of the thin metal film used are important for a highly sensitive detection of viruses. The LSPR-based virus detection technique basically consists of an optimized thin metal film modified on a prism for coupling light. On the contrary, LSPR requires meticulous nanoparticle control technology to detect virus particles because more localized plasmons emit highly strong signal responses. It is also worth mentioning that, as SPR does not require a prism, it is possible to construct a detection system using only a light source and a detector that can irradiate specific light. I have also discussed the elements and developments that are required for the next-generation virus detection technology by describing recent examples of research in SPR and LSPR.

## 2. Current Advancements in SPR-Based Sensor to Detect Virus Particles

### 2.1. Basic Design Method of Virus Detection Technology Based on SPR

**Figure 2:**
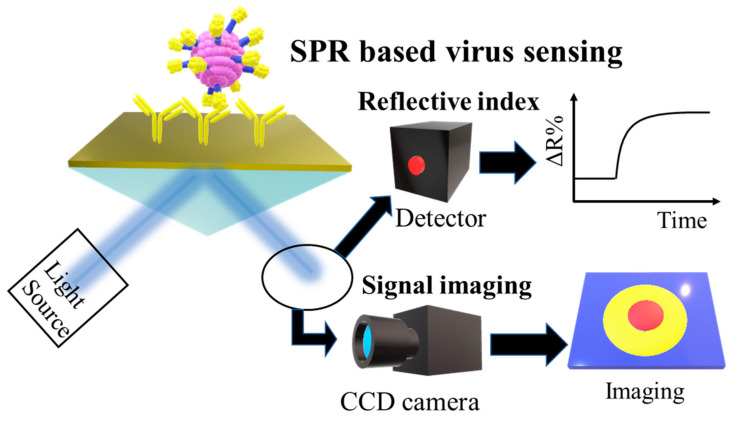
Schematic diagram of SPR-applied virus detection. The reflected light from the light source irradiated on the Kretschmann configuration is captured by the detector or CCD camera and processed and analyzed as a signal.

Metal substrates show strong SPP when irradiated by an appropriate laser [33]. The most typical and effective example based on this phenomenon is the Kretschmann configuration that uses thin films of gold, silver, or a material with other plasmonic properties, such as Pt and Ti, coated on a prism as a substrate for SPR [34]. When optimal light is irradiated, evanescent waves penetrate the thin metal film and cause plasmon excitation on the outer surface of the film. The SPP excitation occurs outside the metal film with the medium. This excited state is sensitive to the changes in the refractive index of the medium, and thus, the effects of objects adsorbed on the medium can be measured with high sensitivity. In the Kretschmann configuration, the thin metal film on the prism that induces SPR and interacts with the light determines the performance of the sensor [35]. Therefore, the thickness of the thin metal film, the material used, and the roughness of the thin film surface are important to consider when designing an SPR-based sensor [36]. Gwon et al. deposited thin gold films of different thicknesses (30, 52, and 70 nm) on a prism and analyzed the reflectance, phase difference, and magnetic field intensity under light irradiation at 632 nm. They found that the 50 nm-thick film provided the most sensitive measurement of the phase difference after light irradiation [37]. The formation of a thin film of optimal thickness on the substrate for SPR is directly related to the sensitivity of the sensor and the reliability of the signal response when applied to virus detection (Figure 2) [38,39].

SPR imaging (SPRi) has also been actively studied as a method to apply SPR in biosensing. SPRi is broadly classified into spectral, intensity, phase, and polarization contrast depending on the signal used [40]. The various optical responses obtained by SPR are captured as images by a CCD camera and analyzed. As a specific application, the interaction of label-free biomolecules bound to a substrate such as a thin metal film in an array format has been reported [41,42]. Proteins with nanoscale, granular, or rod-shaped structures, such as virus proteins, are also easy to measure using SPRi.

### 2.2. Application of Reflection Angle Change by SPR to Virus Detection

In order to effectively utilize the properties of SPR, a method to specifically bind the virus to the metal film is necessary. Antibodies are suitable as trapping materials because they can be modified based on the substrate using chemical cross-linking, and by selecting a structure with high specificity, a highly selective sensor can be easily constructed [43]. Table 1 summarizes the reports on SPR-based virus detection systems that use the antigen–antibody reaction, focusing on the materials used for the thin films, their thickness, and virus detection sensitivity. Some of the references used a two-layer structure with different metal substrates, but the total thickness of the thin films was generally around 50 nm.

The sensitivity of a rapid diagnostic kit for influenza virus (IFV) based on the principle of immunochromatography, which is widely developed as a commercial test using antigen–antibody reactions, was 10^3^ plaque forming unit (PFU)/mL, 10⁴ copies/mL, and 100 pg/mL [44,45,46]. The most basic IFV detection using a thin gold film showed lower sensitivity than the rapid detection kit reported in the case study. However, using a special thin film structure, the virus could be detected with an average sensitivity 100 times higher than that of the existing techniques. From this point of view, it can be considered that the advancement of thin metal films is essential for the practical application of SPR-based virus detection systems. Several reported cases of successful detection of biomaterials using SPR with high sensitivity are attributable to the advancement of thin metal films [47,48,49].

Su et al. and Chang et al. reported a bilayer structure of silver and gold thin films [50,51]. These are examples of how the thin film structure improves the sensitivity of virus detection using SPR. The best feature of this structure is that the silver film, which is an excellent SPR signal-response material but can be easily oxidized, was covered with a gold film to provide an antioxidant effect. However, when gold thin films were formed as ultrathin films (thickness of less than 10 nm), structural changes over time and formation of nanoparticles were reported [52]. The stability in long-term storage is also an important factor for effective virus detection. Although the bilayer structure of a thin metal film improves the sensitivity of the sensor, the advanced structure of the film has some disadvantages. SPR-based virus detection using antigen–antibody reaction is more sensitive than immunochromatographic or ELISA methods. To make this technology more practical, the stability and uniformity of the base and higher sensitivity of the detection method are required.

In recent years, SPR-based virus detection using aptamers, which can bind to the target antigens and antibodies, has been reported [57,58,59]. The use of aptamers is also effective in SPR-based virus detection technology because of the ease of functionality, as many SPR-based platforms use gold, which exhibits high antioxidant properties. Aptamers are promising as basic materials for biosensors because they can be synthesized in large quantities at a low cost once the sequence is determined. In contrast, hybridization of viral DNA/RNA is also required in detecting the virus in an SPR-based system. It has been reported that SPR can also be used to monitor DNA hybridization [60,61]. Therefore, it is possible to detect viruses with high sensitivity by hybridizing viral DNA/RNA with a target DNA/RNA-specific probe modified on the surface of the SPR substrate. In this section, the focus was on virus sensors using antibodies as capture materials. However, the strength of SPR as a sensor principle is that versatile and highly sensitive detection systems using materials that capture the target virus at the interface can be constructed.

### 2.3. Application of SPR Signal Response to Imaging during Virus Detection

Wang et al. reported the successful measurement of IFV with a sensitivity of 1 ag (0.2 fg/mL) using SPRi [62]. The authors used intensity as the signal response of SPR and imaging with a CCD camera to capture the surface plasmon diffusion that occurs when IFV binds to anti-influenza antibodies modified on the substrate surface and accurately detected the mass and size of the virus particles. The IFV particle size is approximately 100 nm, which is relatively large among viruses, while the size of mosquito-borne viruses such as SARS-CoV-2, norovirus, and dengue virus is approximately 30 nm [63,64,65,66]. In SPRi, the molecular weight and particle size of the proteins have a significant effect on the sensitivity. The sensitivity obtained in their study suggests that even a small number of virus particles can be detected. SPRi has high potential as a next-generation virus detection technology. Sun et al. also succeeded in detecting a single T4 phage virus using polystyrene nanoparticles by applying surface plasmon diffusion [67]. Not many examples of effective applications of SPRi for virus detection are presented in this review. However, the principle of SPRi in detecting a single virus particle per measurement is potent. The technical challenge for the next-generation virus detection technology is that it requires more sophisticated equipment compared to the commercially available rapid diagnostic kits [68].

**Figure 3:**
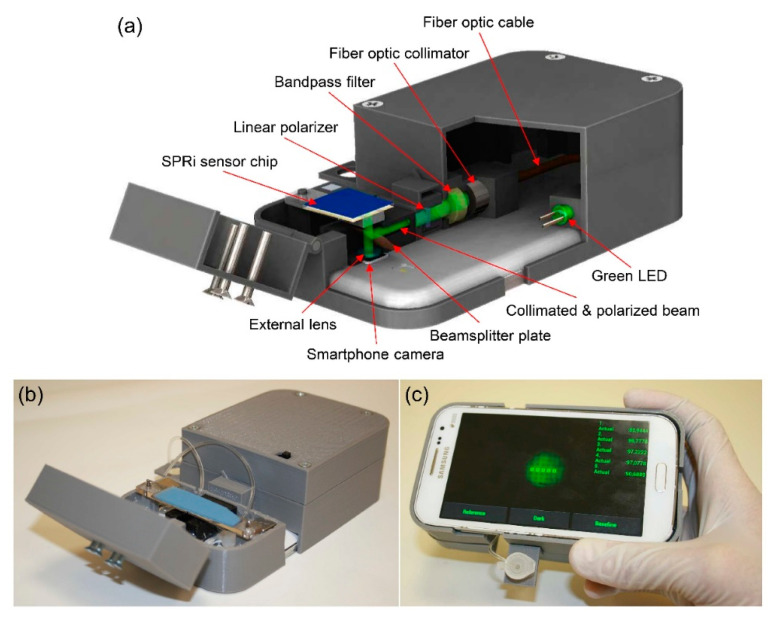
Surface plasmon resonance imaging platform integrated with a smartphone. (**a**) Schematic illustration and (**b**) photograph of the imaging apparatus. (**c**) Custom developed smartphone application for real-time and on-site monitoring of multiple sensing spots. Reprinted permission obtained from [69].

Regarding the improvement of the SPRi device, Guner et al. reported the development of a very efficient system [69]. The authors successfully designed a low-cost SPR-based sensor chip based on the commercially available optical storage disks and developed a compact, smartphone-mountable device using LEDs as the light source (Figure 3). Imaging and detecting antigens captured by the antibodies modified on a thin silver and gold bilayer film were performed. The authors concluded that using a two-layered metallic thin film that can utilize visible light led to the development of a compact and effective biosensing smartphone device. The results also showed that the detection sensitivity was comparable to that of the commercially available SPRi systems, and the authors discussed the future potential of the system as a basis for successful miniaturization and cost reduction.

### 2.4. Current Application of SPR for Virus Sensing

**Figure 4:**
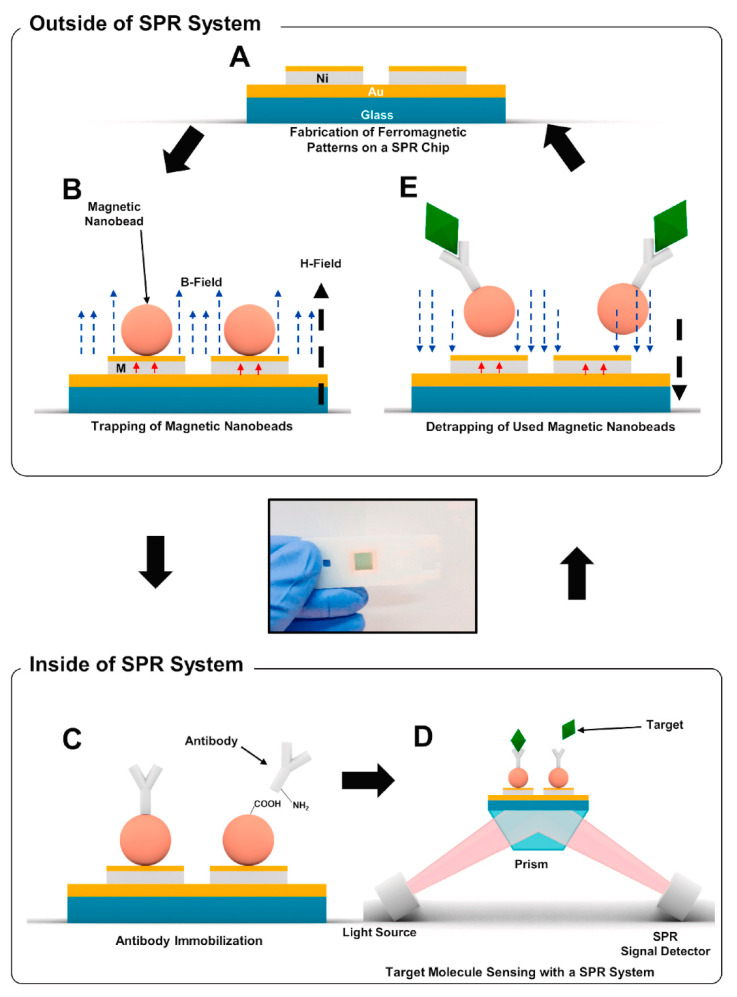
Schematic diagram depicting the cyclic process for the repeated sensing measurements using reusable SPR biosensor chip. (**A**) A reusable SPR chip including ferromagnetic nickel patterns on a conventional SPR chip structure. (**B**) Trapping of magnetic particles on the reusable SPR chip through an external magnetic field. (**C**) Immobilization of antibodies on magnetic particles using EDC-NHS coupling in a conventional SPR system. (**D**) Detection of target molecules. (**E**) Removal of magnetic particles by an external magnetic field in an opposite direction to that for trapping. Reprinted permission obtained from [70].

Up to this section, the potential of SPR and SPRi-based systems as virus detection methods has been discussed. It is also clear that applied research is needed to make the method simple and effective for implementation as a next-generation sensing system. Yoo et al. demonstrated a reusable detection substrate for a new type of SPR-based virus detection system [70]. A system design that allows the SPR platform to be used repeatedly without the need for antibody modifying processes will be a significant step toward the future implementation of virus sensors. In their study, the virus was captured by antibodies conjugated to magnetic beads at specific sites on the substrate (Figure 4). The magnetic nanoparticles were transported by an external magnetic field to the substrate where a gold/nickel (10 nm/50 nm) thin film was patterned on a gold/chromium (45 nm/5 nm) thin film, which was the SPR sensor site (Figure 4A,B). The antibodies were modified on the magnetic nanoparticles at the patterning site by cross-linking the carboxyl groups modified on the magnetic nanoparticles with the amino groups of the antibodies, using a chemical cross-linking agent to provide reactivity with the virus (Figure 4C,D). It was demonstrated that the antibody-modified magnetic nanoparticles bound to antigens could be easily removed from the substrate after virus detection by applying an external magnetic field in the opposite direction of the deposition (Figure 4E). Several studies had already reported that magnetic nanoparticles are suitable materials for signal enhancement in sensing by SPR [71,72]. The contribution of magnetic nanoparticles to the sensitivity is not discussed in this review; however, the compact system has been successfully used for linear, wide-range IFV nucleic protein detection in the range of 300 ng/mL to 10 µg/mL. It was suggested that magnetic nanoparticles show a signal response in this system, at least on the SPR platform. In addition, authors have demonstrated that magnetic nanoparticle removal and detection can be repeated up to seven times on the same substrate, making the author’s idea a more practical SPR-based virus detection technique.

**Figure 5:**
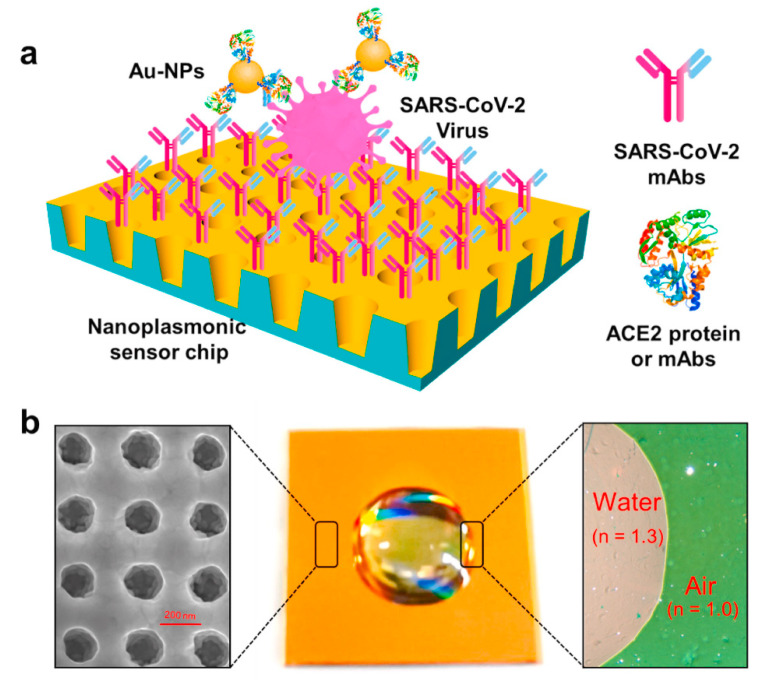
Label-free detection of SARS-CoV-2 pseudovirus with a nanoplasmonic sensor. (**a**) Schematic diagram of the nanoplasmonic resonance sensor for determination of SARS-CoV-2 pseudovirus concentration. (**b**) Photograph (Middle) of one piece of Au nanocup array chip with a drop of water on top. Scanning electron microscopy image (Left) shows the replicated nanocup array. Transmission microscopy image (Right) shows that air and water on the device surface exhibit different colors, green and far-red pink, respectively. Reprinted permission obtained from [73].

Huang et al. applied their low-cost plasmon nanoarray SPR chip design to detect SARS-CoV-2 (Figure 5a) [73,74,75]. The structural characteristics of the substrate included the uniform and tight formation of a special nanosized cup structure, which made it possible to observe the plasmon resonance wavelength and intensity changes without the need for external optics because of the extraordinary optical transmission [76]. A further feature is that gold nanoparticles are functionalized with ACE2 protein to bind SARS-CoV-2. It has been reported that the sandwich structures of SPR substrate-virus particles-gold nanoparticles enhance the SPR signal response [77]. In this research, high sensitivity was achieved by applying the characteristics of the gold thin film structure and metal nanoparticles rather than using a metal bilayer structure. An important aspect of the application of thin films with such special structures is to maintain a uniform surface structure. The uniformity of the gold-nanoparticle cup fabricated substrate was observed by scanning electron microscopy, and it was demonstrated that water drops on the substrate showed different color from those in the air (Figure 5b). The authors noted that the characteristic spectral changes made it possible to detect the virus by SPR with high sensitivity.

**Figure 6:**
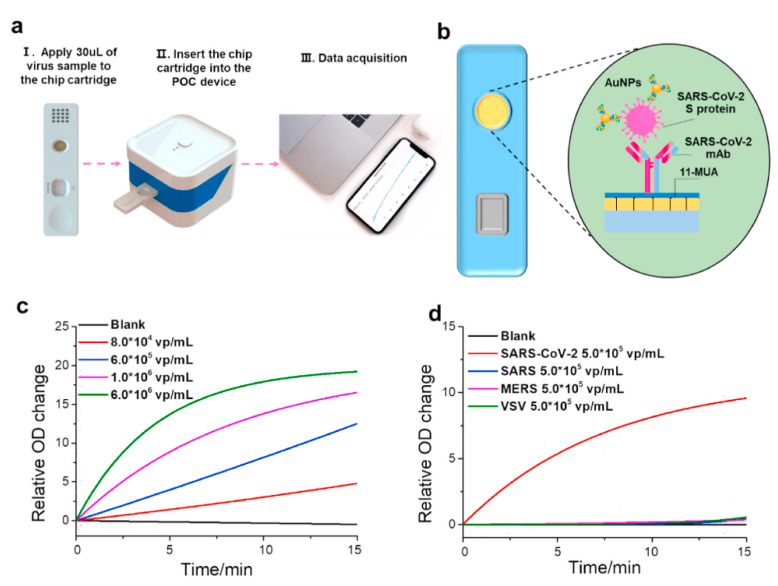
Detection of SARS-CoV-2 pseudovirus with nanoplasmonic sensor chips by a point-of-care device. (**a**) Schematic of nanoplasmonic sensor chip cartridge detecting SARS-CoV-2 pseudovirus with a low-cost handheld point-of-care testing device. (**b**) The illustration shows the detection process of the sensor chip cartridge for specific SARS-CoV-2 detection. (**c**) Dynamic binding curves of virus and antibody interaction with different concentrations of the SARS-CoV-2 pseudovirus over the range 0–6.0 × 106 virus particles (vp)/mL at the resonance wavelength. (**d**) Specificity verification test: Dynamic binding curves of SARS-CoV-2 antibodies interaction with different pseudovirus of SARS-CoV-2, SARS, MERS, and VSV at the concentration of 5.0 × 105 vp/mL. Reprinted with permission obtained from [73].

The SPR-based sensor chip reported in this study could be incorporated into a 96-well plate or cartridge [73]. This section focuses especially on the cartridge-type detection systems that can be used for on-site detection and other applications. The cartridge SPR system controlled by a smartphone application could measure the dynamic absorption spectra by simply inserting the antibody-modified SPR nanocup placed on a dedicated cartridge (Figure 6a,b). The SPR-based sensor chip site was chemically cross-linked with virus-specific antibodies on a nanocup structure coated with gold thin film to provide reactivity with SARS-CoV-2. The detection results presented by the authors demonstrated that the SARS-CoV-2 concentration-dependent signal response in this transportable SARS-CoV-2 reservoir was highly selective, with no signal response specific to other virus species. Furthermore, the lower limit of quantitative detection was set at approximately 4000 viruses, and it is expected that the lower limit of quantitative detection will be the same as that of a large device with further sensitivity improvements (Figure 6c,d). In this study, the authors chemically cross-linked antibodies to the substrate by forming a self-assembled monolayer using 11-mercapto-undecanoic acid (11-MUA) for antibody modification. This is a well-known chemical cross-linking method, and it has succeeded in yielding highly efficient antibody modification on the surface [78,79]. However, one area that remains controversial is the randomness of the antibody conjugation sites. Since the gold film had a nanocup structure with a diameter of more than 200 nm, the surface modification by 11-MUA was likely carried out uniformly into the cup. It is unlikely that antibodies, which are structures smaller than 10–15 nm, bind only to the surface layer of the gold nanocups [80]. The signal generated by the binding of the virus particle to the antibody modified in the cup might be different from the signal generated by the binding of the virus to the antibody conjugated on the top surface; as the sensitivity of the detection system increases, the magnitude of randomness caused by such antibody modification methods increases [81]. Therefore, modifying antibodies only at selective sites is important to minimize the difference in signal response between virus detection sites when the substrate forms a complex shape. Site-specific conjugation of the antibody can be achieved only under special conditions. There is no report about site-specific conjugation of antibodies on a single material-based nanostructural substrate, which requires further innovation to fabricate the next-generation SPR-based virus sensors.

In this section, two different types of currently reported SPR sensors for virus detection were introduced. The common thread among the studies was developing a more practical design of SPR-based virus detection systems with a certain level of sensitivity. The research focus in recent years on virus detection technologies has often been on sensitivity and speed, and for on-site detection technologies, whether the system can be handheld or wireless and employs reusable methods [82,83,84,85,86]. As for the research on virus detection technology using SPR, the major points required in the future are not high sensitivity and rapidity, but thin film formation and suitable construction systems to enhance the practicality and applicability.

## 3. LSPR Phenomenon on Nanoscale Systems and Application of LSPR to Virus Sensing

### 3.1. Signal Response Produced by LSPR for Virus Detection

**Figure 7:**
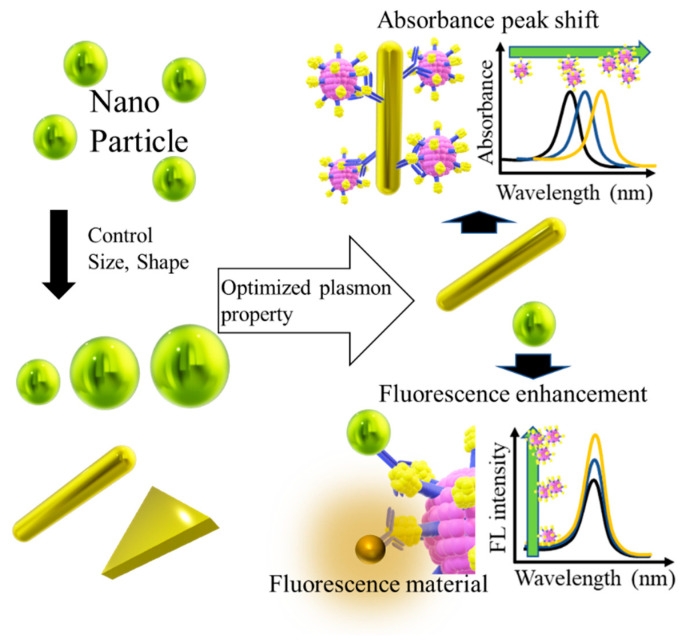
Basic development method of virus detection technology based on LSPR. Optimal materials with the desired plasmonic properties are obtained by controlling the shape and size of the nanoparticles. The optimal material is capable of generating a sensitive signal response in the presence of a virus.

After LSPR, an enhancement of electrolysis and light quenching occurs in the vicinity of the nanoparticles, based on the size, shape, and material used [87,88,89]. Therefore, the size and shape of the nanoparticles need to be precisely controlled to obtain the desired optical response. The most fundamental application of optical signal response by LSPR is the change in the peak absorption wavelength due to the local refractive index change on the nanoparticle surface (plasmon peak shift) and the fluorescence enhancement effect caused by using near-field light. (Figure 7). In this section, the focus is on virus detection techniques that use the basic optical response of LSPR. Further, the two types of responses that are still being applied to virus detection are summarized in this section.

LSPR is adapted for transmission measurements, whereas SPR can measure the reflectance change when some material is attached to a single particle [90,91,92]. The advantage of the former is that the measurement system can be simply constructed without the need for movable elements necessary for reflection angle measurement [93]. The transmissive signal response has already been applied in various virus detection applications as the plasmon peak shift of nanoparticles sensor compared with an LSPR-based reflective signal sensor [94].

The near-field light formed near the noble metal nanoparticles by LSPR enhances the interaction of the light with the fluorescent material present at a certain distance in the region [95,96,97]. The resulting significant fluorescence enhancement effect of the fluorophores is widely used in the development of highly sensitive sensing techniques, as the effect enables the emittance of signal responses even from small amounts of sample. These observations render LSPR a potential signal amplification method for virus sensing [98].

### 3.2. Optical Absorbance Peak Shift Application for LSPR-Based Virus Sensing

**Figure 8:**
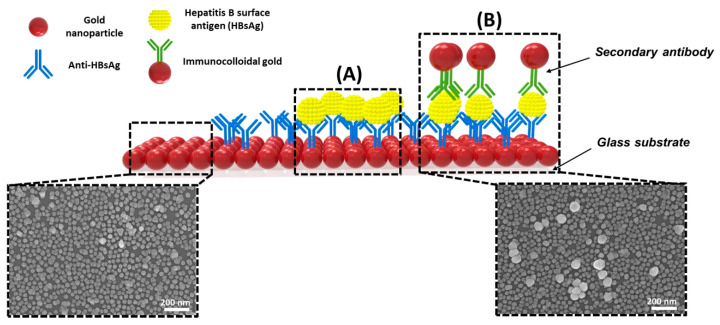
Detection of HBsAg by single assay LSPR sensing chip format (**A**) and modified heteroassembled AuNP sandwich immunoassay LSPR chip format using immunocolloid AuNPs (**B**). Changes in the spectrum peak at different concentrations of HBsAg (1 pg/mL to 1 µg/mL HBsAg), reacted on the chip. Insert image is presenting the immunological reaction occurring on the active site of LSPR sensing chip. 15, 30 and 50 nm of immunocolloid AuNPs as signal enhancers. 1 ng/mL to 100 fg/mL HBsAg reacted the LSPR chip. All experiments were conducted in six measurements, and data represent the mean ± standard deviation. The coefficient of variation (% CV) is below 10%. Reprinted with permission obtained from [101].

To measure the local refractive index change as an optical response at the nanoscale level, the strategy used for constructing LSPR sensors is similar to that of SPR sensors. It is important to modify the material to capture the target on the nanomaterials with high efficiency while maintaining the structural properties of the nanoparticles [99]. The shift in plasmon peak value due to the LSPR of plasmon particles after virus binding is also a sensitive signal response [100]. Kim et al. designed a structure in which a virus was sandwiched between two different sizes of AuNP on an AuNP-laden substrate (Figure 8) [101]. In this method, two gold nanoparticles in close proximity repel each other in a plasmon resonance state in the presence of the virus, resulting in a stronger peak shift effect than that in the non-sandwich state. The signal enhancement effect of the sandwich structure has been demonstrated, and a 100-fold increase in the sensitivity has been successfully achieved compared to the virus captured by AuNP-laden substrate only. Furthermore, the sensitivity of the sandwich structure differed depending on the size of the gold nanoparticles on the secondary antibody site. The smaller the particle, the stronger the signal response, even at low sample concentrations. This work demonstrated that particle size has a significant effect on the use of LSPR for developing application-based systems.

There are other techniques of virus detection based on absorbance measurement and nanoparticles, such as the agglomeration of nanoparticles in the presence of a target [102,103]. The peak change of the plasmon band was measured for the structural change due to the aggregation of particles [103]. In this section, the optical response analogous to SPR and the plasmon peak shift due to local plasmon response on the nanoscale caused by the binding of nanoparticles to target materials were introduced.

### 3.3. LSPR Fluorescence Enhancement for Virus Detection

**Figure 9:**
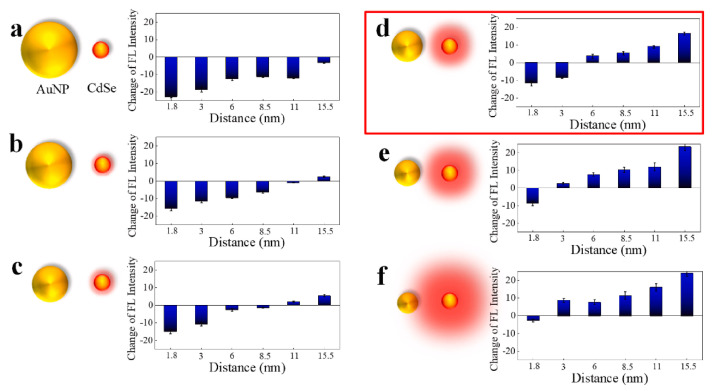
Fluorescence spectral bar diagram of CdSe QDs in various CdSe QD-peptide-AuNP nanoconjugates. Six different sizes of AuNPs at 80 (**a**), 60 (**b**), 45 (**c**), 35 (**d**), 25 (**e**), and 15 nm (**f**) combined with six different peptide lengths produced various fluorescence intensity changes. Reprinted with permission obtained from [104].

Electric field enhancement around the nanoparticles by LSPR produces near-field light, which can enhance fluorescence by improving the interaction of light with the fluorescent materials placed at an appropriate distance. The distance between the plasmon particle and the target fluorescent material is the most important factor in the fluorescence enhancement effect of LSPR. Chowdhuly et al. used gold nanoparticles as a plasmon amplifier and quantum dots (QDs) as fluorescence materials to analyze the fluorescence enhancement effect of LSPR on fluorescent materials with respect to plasmon particle size and interparticle distance (Figure 9a–f) [104]. The fluorescence of QDs placed by chemical cross-linking in the vicinity of gold nanoparticles, with particle sizes ranging from 15 to 30 nm, proved to be fluorescence-enhancing for an interparticle distance of up to 15.5 nm, except for the nearest neighbor distance of 1.8 nm. The fluorescence enhancement effect decreased with distance and as the size of the gold nanoparticles approached 100 nm (micro-order), the effect was replaced by a strong trend of quenching effect of the QDs (Figure 9a–f). Their results showed the distance to be maintained between the electromagnetic fields formed in the vicinity of the plasmon particle by LSPR and the fluorescent material.

Many studies have reported the application of the fluorescence enhancement effect of LSPR for virus detection. Nasrin et al. recently reported a nanocomposite in which the plasmon particles and QDs were combined by chemical cross-linking and modified with antibodies for supplementation, thereby providing reactivity against viruses [105]. The distance between the particles was designed to be optimal for fluorescence enhancement through antigen–antibody reaction on the virus. The virus detection techniques based on the fluorescence enhancement effect of fluorescent materials by LSPR are summarized in Table 2, focusing on the sensitivity of the technique, the materials used, and whether the particle distance was controlled precisely or roughly. Even when using gold nanoparticles, which are the most basic plasmon particles, a 10-fold difference in sensitivity was obtained depending on whether the distance to the fluorescent material was accurately controlled [106,107]. In addition, a sensor designed for Zika virus RNA successfully detected even a single viral RNA molecule by maintaining a constant interparticle distance through chemical bonding. The interparticle distance was shown to significantly affect the sensitivity of the LSPR fluorescence-enhanced virus detection technique [98]. As for the structure of the plasmon particles used, particles with a bimetallic structure and composite nanomaterials showed very high sensitivity. Hence, the structure of composite nanomaterials is likely to render more sensitive LSPR and SPR responses. The most striking example is a composite of magnetic nanoparticles, gold nanoparticles, and graphene, which successfully detected IFV from an ultra-low concentration of 7.27 fg/mL. In this study, the authors not only enhanced the LSPR effect by complexing with graphene, but also achieved direct virus detection in clinical specimens containing high levels of contaminants by magnetic separation of plasmonic nanoparticles [108].

The LSPR-based fluorescence virus sensor should have a controlled nanoscale distance between the plasmon particles and fluorescence material. In the case of antigen–antibody reaction, forming a thin film on the plasmon particles beforehand to control the nanometer distance is effective [111]. A typical example of functionalization of nanomaterials is the self-assembled monolayer of the layer-by-layer method [112,113,114]. Both methods essentially require a thin film of desired thickness and the modification of antibodies and aptamers. When virus-specific DNA/RNA is used as a probe, the length of the sequence during hybridization can be easily adjusted. Therefore, it is only necessary to functionalize the probe directly on the plasmon particle [115,116]. The development of virus detection technology based on the LSPR fluorescence enhancement effect is challenging because it requires precise control of the nanoparticles’ size and particle distance to detect viruses with higher sensitivity. However, developing a simple and reproducible control method will be a necessary element for the next generation of LSPR detection technology, as all the reported studies have succeeded in detecting viruses with high sensitivity.

## 4. Conclusions and Future Prospects

SPR and LSPR-based virus sensors amplify the signal response of samples at extremely low concentrations, which is otherwise difficult to read and analyze using conventional methods. Commercially available virus detection systems, except PCR, are easy to use and can process multiple samples but have considerable sensitivity limitations, resulting in false negatives. The detection methods based on SPR and LSPR require only virus capturing at the sensor site, rendering the methods simple and highly sensitive. To achieve high sensitivity, the structure and thickness of the thin metal film must be optimized for the SPR-based sensor, and the size and shape of the plasmon particles must be controlled for the LSPR-based sensor. In addition, the SPR-based sensors are witnessing advancements with respect to reusability and miniaturization. As for LSPR, it is easy to construct a simple system to observe the optical response effectively. Collectively, studies on SPR and LSPR-based virus sensing research have already demonstrated sufficient sensitivity. Table 3 summarizes the respective characteristics of SPR and LSPR. It can be seen that each principle has high potential for practical use as a detection technology. In particular, LSPR is more advantageous for miniaturization because it requires only a light source and a detector as the device configuration. Therefore, as with the SPR and LSPR system, more practical developmental research will continue to progress in the future. The next-generation virus detection technology should combine simplicity and rapidity with high sensitivity. For simplicity, only one step and mixing of solutions is required to constitute a system that can be implemented without specialized knowledge. Rapidity can be improved by searching for optimal modification methods for supplementary substances and by applying novel substances such as aptamers. In addition, it should detect the target virus irrespective of the measuring environment and without being affected by the presence of foreign substances. Social implementation of SPR or LSPR virus detection sensors that combines these elements will allow personnel to perform rapid virus detection in airports in one person/two minutes using a combination of a smartphone and a simple detection kit. Real-time, on-site detection is a technology that has not yet been achieved and will be required in the future, and further development of SPR and LSPR-based virus detection technology that can achieve this is expected.

## Figures and Tables

**Table 1:** Comparison layer material and thickness related with limit of detection (LOD) for virus sensing using SPR.

Layer Structure	Thickness of Layer	Target Virus *	LOD	References
Gold thin film	~50 nm	IFV	193.3 ng/mL	[53]
Gold thin film	~50 nm	DV	0.08 pM	[54]
Gold thin film	50 nm	EBoV	0.5 pg/mL	[55]
Gold/Silver thin film	8/37 nm	IFV	30 PFU/mL	[50]
Gold/Silver thin film	10/35 nm	IFV	144 copies/mL	[51]
Platinum-di-selenide/Gold thin film	2/48 nm	COVID-19	1.95 nM	[56]

* IFV: Influenza virus, DV: Dengue virus, EBoV: Ebola virus.

**Table 2:** Comparison of sensitivity of techniques applying the fluorescence enhancement effect of LSPR of plasmonic particles to virus detection.

Plasmon Particle	Fluorescence Material	Particle Distance	Target Virus	Target Material	LoD	Reference
AuNP	QD	roughly controlled	IFV	Antigen	0.4 pg/mL	[106]
AuNP	QD	Precisely controlled	NoV **	Antigen	12.1 fg/mL	[107]
AuAgNP	QD	Precisely controlled	ZIKV	RNA	1.7 copies/mL	[98]
AuNP	Fluorescence Dye	Precisely controlled	IFV	Antigen	1 pM	[109]
AgNP *	Fluorescence Molecule	roughly controlled	IFV	Antigen	2 ng/mL	[110]
AuNP-magnetic nanoparticle-graphene	QD	roughly controlled	IFV	Antigen	7.27 fg/mL	[108]

* AgNP = Silver nanoparticle, ** NoV = Norovirus, ZIKV = Zika virus.

**Table 3:** Differences between SPR and LSPR in virus detection technology development.

	SPR	LSPR
Scale	Micro Scale (Film)	Nano Scale (Particle)
System complexity	Need prism to couple the light	Only light source
electromagnetic field decay length	Long	Short
Signal sensitivity for virus	Sensitive	Sensitive
Signal stability	Bulk on the film effected	Depends on stability of particle
Thermal control	Needed	No need
Reproducibility of material	Easy	Depends on material
Miniaturization of devices	Prism make limitation	Only light source

## Data Availability

Data are contained within the article.
